# Beyond the autonomous individual: hydrosocial relations and the constitution of the relational hydrosocial self in an Andean conflict

**DOI:** 10.3389/fsoc.2026.1856916

**Published:** 2026-09-07

**Authors:** Saríah Fanny Oré Gálvez, Alex Foyams Molina Linares, Jherry Lobaton Phocco Minaya, Alex Quispe Quispe, Solón Dante Carhuallanqui Ibarra, Juan Carlos Terres León, Rubén Ñaupari Molina, Crispin H. W. Barnes, Luis De Los Santos Valladares

**Affiliations:** 1 Escuela Profesional de Ingeniería Ambiental, Universidad Nacional Autónoma de Huanta, Ayacucho, Peru; 2 Escuela Profesional de Ingeniería Topográfica y Agrimensura, Facultad de Ciencias Agrarias Universidad Nacional del Altiplano Puno, Puno, Peru; 3 Escuela Profesional de Educación, Especialidad de Ciencias Sociales, Universidad Nacional de San Antonio Abad del Cusco, Cusco, Peru; 4 Universidad Nacional José María Arguedas, Andahuaylas, Peru; 5 Cavendish Laboratory, Department of Physics, University of Cambridge, Cambridge, United Kingdom

**Keywords:** Andean studies, distributed agency, hydrosocial territory, moral ecology, ontology, relational hydrosocial self, socio-environmental conflict, territorial subjectivity

## Abstract

This article addresses a specific under-theorized problem in social theory: how subjectivity is constituted through relations in which ecological and nonhuman entities are not contextual but constitutive. Against dominant models that define the self as autonomous, bounded, and human-centered, it argues that subject formation can emerge through hydrosocial relations linking humans, water systems, territory, and nonhuman entities. Drawing on qualitative fieldwork in the Razuhuillca micro-watershed in the Peruvian Andes, the study combines in-depth interviews, walking ethnography, field observation, visual documentation, and complementary lexical analysis. The findings suggest that, in this case, water is understood not as a resource external to society but as a living condition of existence embedded in moral, spiritual, and territorial relations. Agency is distributed across humans and nonhumans, particularly Apu Razuhuillca, while ritual practices, collective care, and environmental narratives sustain a morally embedded hydrosocial field. The analysis identifies five mechanisms through which subjectivity is constituted: discursive enactment, distributed agency, moral embedding, experiential validation, and territorial practice. Together, these mechanisms provide a concrete account of how relational configurations are translated into forms of selfhood. On this basis, the article proposes the concept of the relational hydrosocial self as an analytical specification of how identity, agency, and moral responsibility emerge through hydrosocial relations. Rather than introducing a wholly new theory, the concept offers a more precise account of a limitation shared across relational sociology, political ontology, and more-than-human approaches by showing how ecological and nonhuman relations become constitutive of subject formation. In doing so, the article extends relational theories of the self beyond human-centered sociality, advances political ontology by linking ontological plurality to subject formation, and contributes to hydrosocial theory by suggesting that, in this case, water relations shape not only territory and governance, but also the constitution of the self.

## 1 Introduction

The concept of the self has occupied a central yet contested position within social theory. Classical sociological traditions largely framed the individual as the primary locus of agency, rationality, and social action, reflecting philosophical inheritances rooted in Western modernity that conceptualize the subject as autonomous and bounded (Taylor, 1989; Giddens, 1984). Within this framework, society appears either as the aggregate outcome of individual actions or as a structure constraining otherwise independent actors, reproducing the enduring tension between methodological individualism and structural determinism. Despite successive theoretical attempts to mediate this divide, including structuration theory (Giddens, 1984), habitus theory (Bourdieu, 1977), and communicative action (Habermas, 1987), dominant conceptions of the self in social theory continue to presuppose a separation between human subjects and the material environments they inhabit.

In recent decades, this assumption has been increasingly challenged by relational, ontological, and more-than-human approaches that question the universality of the modern individual. Relational sociology argues that actors emerge through networks of interaction rather than pre-existing entities (Emirbayer, 1997; Cardenas Morales et al., 2025a), while political ontology and decolonial scholarship emphasize that different collectives enact distinct realities in which humans, nonhumans, and territories participate jointly in social existence (Escobar, 2018; Blaser, 2013). Relatedly, more-than-human approaches have shown how agency is distributed across human and nonhuman entities rather than concentrated exclusively in human actors. Together, these perspectives challenge the autonomous, bounded, and human-centered self.

Yet an important gap remains. While these literatures have said much about relational actors, ontological plurality, and distributed agency, they have been less explicit about how subjectivity itself is constituted through relations in which ecological processes and nonhuman entities are not external context, but constitutive elements of social life. In other words, existing scholarship has decentered the autonomous individual, but has less often specified how the self is formed within relational configurations that include water, territory, and nonhuman beings as active participants.

This gap becomes especially visible in contexts of socio-environmental conflict. Environmental disputes are not only struggles over resources or governance arrangements, but also sites where different forms of life, responsibility, and subjectivity are enacted and contested. Research on hydrosocial territories has shown that water conflicts often express competing ontologies of human–environment relations (Boelens et al., 2016; Budds and Hinojosa, 2012). In many Andean contexts, water is understood not as a resource external to society, but as a living relational entity embedded within moral, spiritual, and territorial orders (Ulloa, 2017). Such configurations challenge modern distinctions between subject and object, revealing forms of agency distributed across humans, landscapes, and cosmological entities. Yet these dynamics have often been approached as cultural meanings or belief systems rather than as constitutive dimensions of subject formation.

This article addresses that problem by examining how hydrosocial relations participate in the constitution of subjectivity. It draws on empirical material from the Razuhuillca micro-watershed conflict in the Peruvian Andes, where local communities describe water as “the blood of the mountain” and invoke Apu Razuhuillca as a territorial guardian. Building on previous research that documented hydrosocial vulnerability and territorial resistance in this context (Cardenas Morales et al., 2025b, 2026), the analysis shifts the focus toward subject formation as an emergent effect of hydrosocial relations.

Rather than interpreting these expressions as symbolic metaphors alone, the article approaches them as ontological statements that organize action, responsibility, and collective agency. Following Ricoeur's (1992) notion of narrative identity and Taylor's (1989) account of morally embedded selves, the analysis explores how subjectivity is produced through relational attachments linking community, territory, and water. In this sense, discourse is treated not merely as representation, but as a world-making practice (Bruner, 1991; Somers, 1994) through which relational configurations are enacted and sustained.

The main contribution of this article is to specify how subjectivity may be understood as hydrosocially constituted. It does so by identifying a limitation shared across relational sociology, political ontology, and more-than-human approaches: although these perspectives have demonstrated relationality, plurality, and distributed agency, they have been less explicit about how these dimensions become integrated into the formation of the self. To address this limitation, the article proposes the concept of the relational hydrosocial self, understood as an analytical account of how identity, agency, and moral responsibility emerge through relations linking humans, water systems, territory, and nonhuman entities.

Rather than proposing a wholly new theoretical paradigm, the concept offers a specification of the mechanisms through which relational, ecological, and cosmological dimensions become internal to subject formation. Grounded in empirical evidence from an Andean hydrosocial conflict, the article contributes to relational sociology by extending subject formation beyond human-centered interaction, advances political ontology by linking ontological plurality to the production of selfhood, and contributes to hydrosocial theory by suggesting that water relations shape not only territory and governance, but also the constitution of subjectivity. In this way, the relational hydrosocial self provides a framework for rethinking the self as a distributed, morally embedded, and territorially enacted process within socio-ecological life.

## 2 Materials and methods

### 2.1 Research design

This study adopts a qualitative, relational ethnographic research design to examine how subjectivity is constituted through hydrosocial relations in the context of socio-environmental conflict. The research is grounded in a relational ontology that conceptualizes the self as emerging through interactions among humans, water systems, and territorial entities. Methodologically, this perspective is operationalized through an integrated analysis of discourse, practice, and lived experience, allowing for the examination of subjectivity as a relational and processual phenomenon.

The empirical focus is the Razuhuillca micro-watershed in the Peruvian Andes, a hydrosocial landscape characterized by the coexistence of ecological processes, ritual practices, and ongoing territorial conflict. Previous studies in this context have examined hydrosocial resilience, territorial disputes, and governance dynamics. Building on this body of work, the present study shifts the analytical focus toward the constitution of subjectivity, examining how relational engagements with water, territory, and nonhuman entities give rise to specific forms of the social self.

To address this question, the research employs a qualitative case study strategy oriented toward analytical generalization rather than statistical representativeness. The objective is not to produce population-level inference, but to identify recurrent relational patterns across cases through in-depth qualitative evidence. This approach is particularly appropriate for contexts in which human–environment relations are embedded within moral, spiritual, and ontological frameworks that cannot be adequately captured through purely quantitative methods.

Participant selection followed a purposive sampling strategy based on three criteria: (i) direct involvement in water-related practices (e.g., irrigation, livestock management, territorial care), (ii) long-term residence in the watershed, and (iii) variation in age, gender, and degree of engagement with hydrosocial relations. This strategy aimed to capture diversity in experiential and ontological positioning rather than statistical representativeness. The characteristics of the interview sample are detailed in Supplementary Table S1.

Hydrosocial conflict is conceptualized not only as a struggle over resources or governance arrangements, but as an empirical setting in which subjectivities are actively produced, negotiated, and contested. In this sense, conflict is treated as an analytical entry point that makes visible how identities, responsibilities, and forms of agency are articulated through relations with water, territory, and nonhuman entities such as Apu Razuhuillca.

The research design is structured to capture three interconnected dimensions of subject formation: discursive enactment, experiential validation, and territorial practice. These dimensions guided both data collection and analysis, allowing for the systematic examination of how language, lived experience, and everyday practices contribute to the constitution of the relational hydrosocial self.

Data collection proceeded iteratively until thematic saturation was reached, understood as the point at which no substantially new relational patterns emerged across interviews. The study adopts a relational ethnographic stance, combining situated engagement with participants and analytical reflexivity regarding the researcher's positionality as an external observer within the field.

### 2.2 Data collection

Data were collected through qualitative fieldwork conducted in the Razuhuillca micro-watershed (Huanta, Ayacucho, Peru) in April 2026. The research combined in-depth interviews, walking ethnography, field observations, and visual documentation in order to capture the relational dynamics through which water, territory, and subjectivity are intertwined.

A total of 12 semi-structured interviews were conducted with local actors, including farmers, livestock keepers, long-term residents, and community members directly engaged in water use and territorial practices. Participants were selected through purposive sampling based on three criteria: (i) direct involvement in water-related practices (e.g., irrigation, livestock management, territorial care), (ii) long-term residence in the watershed, and (iii) variation in age, gender, and degree of engagement with hydrosocial relations. This strategy was designed to capture diversity in experiential and ontological positioning rather than statistical representativeness. Detailed sample characteristics are provided in Supplementary Table S1.

Interviews lasted between 45 min and 90 min and were conducted using an open-ended protocol designed to elicit narrative accounts of participants' relationships with water, the Razuhuillca mountain, and Apu Razuhuillca as a territorial entity. Questions focused on lived experience, interpretation of environmental processes, and forms of engagement with water and territory, encouraging participants to articulate relational understandings rather than abstract opinions.

Interviews were conducted in Spanish and Quechua. When interviews were conducted in Quechua, they were translated into Spanish during transcription through contextual translation, prioritizing semantic meaning over literal equivalence. Particular attention was paid to preserving culturally specific expressions related to water, territory, and nonhuman agency. In cases where key terms did not have direct equivalents, original expressions were retained and analytically interpreted in context.

In addition to stationary interviews, the research incorporated walking ethnography, in which participants guided the researcher through key sites such as springs (puquiales), lagoons, irrigation channels, and high-altitude areas of the mountain. These situated interactions enabled the co-production of narratives in direct relation to the landscape, allowing observation of how meaning, practice, and environment are articulated in context. This approach also facilitated access to experiential knowledge that may not emerge in conventional interview settings.

Field notes were systematically recorded throughout the research process, documenting observations, contextual details, and verbatim expressions considered analytically relevant. Particular attention was given to recurring linguistic expressions, references to nonhuman agency, and accounts of moral or spiritual interactions with water and territory.

Visual data were collected through georeferenced photographs of water sources, landscape features, and everyday practices associated with water use. These images were accompanied by contextual annotations describing location, participants, and locally attributed meanings, enabling the integration of material and symbolic dimensions in the analysis.

All interviews were audio-recorded with prior informed consent. Participants were informed about the objectives of the study, the voluntary nature of their participation, and the anonymization of their data. All data were anonymized and handled in accordance with established ethical research standards for qualitative fieldwork.

The combination of interviews, embodied observation, and visual documentation generated a multi-layered dataset capable of capturing discourse, practice, and lived experience as interconnected dimensions of hydrosocial relations. This triangulated approach enhances the analytical robustness of the study by allowing cross-validation of relational patterns across different forms of evidence.

### 2.3 Analytical strategy

The analytical strategy followed a qualitative, theory-informed and abductive approach combining thematic coding, narrative analysis, and complementary lexical examination to identify patterns in the constitution of relational subjectivity. The analysis moved iteratively between empirical material and theoretical concepts, allowing the refinement of analytical categories and the development of a conceptually grounded interpretation.

First, all interview transcripts and field notes were subjected to an iterative coding process. An initial phase of open coding identified recurrent expressions related to water, territory, and agency. These were subsequently grouped into higher-order analytical categories reflecting key dimensions of hydrosocial relations, including: (i) water as life and condition of existence, (ii) total dependence on water systems, (iii) attribution of agency to nonhuman entities (Apu, mountain, lagoon), (iv) moral regulation through respect, reciprocity, and punishment, and (v) spiritual-territorial practices such as rituals and offerings. The full codebook is provided in Supplementary Table S2.

Through a process of constant comparison across interviews, these categories were further refined and analytically integrated into broader mechanisms of subject formation. This step involved moving from descriptive codes to relational patterns by examining how different categories co-occurred, reinforced each other, and were articulated within participants' accounts. Mechanisms were defined as recurrent relational configurations linking discourse, practice, and experience, rather than as isolated variables.

Second, a narrative analysis was conducted to examine how these coded elements were embedded within broader stories and experiential accounts. Particular attention was given to narratives involving mythical entities, environmental events, and personal or collective experiences interpreted through relational frameworks. Rather than treating such accounts as symbolic representations, the analysis approached them as performative and ontological statements that organize meaning, action, and responsibility within the community.

To strengthen analytical robustness, the study incorporated a limited frequency-based coding component. The occurrence of each thematic category was counted across interviews to identify recurrent patterns and assess their distribution within the dataset. Given the small sample size (*n* = 12), these counts are not interpreted statistically but analytically, as indicators of pattern consistency and salience across cases. Their function is to support qualitative interpretation by distinguishing between isolated references and broadly shared relational expressions.

Additionally, a basic lexical analysis was conducted to examine the frequency and co-occurrence of key terms such as “water,” “life,” “Apu,” and “mountain.” This step was implemented using standard text-processing procedures and served as a complementary validation strategy. Rather than constituting an independent analytical method, lexical analysis was used to corroborate the consistency of associations identified through coding, particularly those linking water to life, agency, and moral relations (Supplementary Tables S3, S4).

Building on these procedures, the analysis identified five interrelated mechanisms of relational self-formation, which were analytically synthesized into three integrative dimensions: discursive enactment, experiential validation, and territorial practice. This synthesis involved examining how mechanisms clustered across cases and contributed jointly to the constitution of subjectivity, rather than treating them as discrete or additive factors.

Variation across participants was explicitly examined to avoid assumptions of cultural homogeneity. Differences in belief, ranging from strong ontological commitment to skepticism, were analyzed as variations within the relational field rather than as deviations from it. This allowed the identification of differentiated pathways of subject formation and supported a non-essentialist interpretation of relational subjectivity.

Data collection and analysis proceeded iteratively until thematic saturation was reached, defined as the point at which no substantively new categories or relational patterns emerged from additional coding.

Overall, this analytical strategy treats discourse not as mere representation, but as a performative practice through which hydrosocial relations and subjectivities are enacted. By combining interpretive depth with systematic pattern identification, the study provides an empirically grounded basis for theorizing the relational hydrosocial self.

## 3 Results

### 3.1 Hydrosocial relations as conditions of existence

The empirical material indicates that water is not conceived as a mere resource but as a fundamental condition of existence embedded within a relational field linking humans, territory, and nonhuman entities. Across interviews, the association between water and life emerged as a highly recurrent expression, appearing in 11 out of 12 cases (Table 1). Participants consistently described water as indispensable not only for agricultural production and livestock, but for the continuity of life itself. Expressions such as “water is life” operate not simply as descriptive statements, but as ontologically meaningful formulations that situate existence within hydrosocial relations.

**Table 1:** Distribution of relational subjectivity codes across interviews.

Code	Description	Frequency (*n* = 12)	Percentage (%)
Water as life	Water is described as a condition of existence for humans, animals, and agriculture	11	91.7
Total dependence	Community survival explicitly depends on water availability	10	83.3
Non-human agency (Apu)	The mountain/Apu is described as an active agent controlling water	9	75.0
Moral regulation	Water scarcity or abundance linked to respect, offense, or ritual practice	8	66.7
Spiritual-territorial relation	Water and territory linked to sacred practices (pagapu, rituals)	7	58.3
Ontological narratives	Presence of myths (sirens, sacred animals, enchanted entities) shaping water relations	8	66.7
Heterogeneous belief	Variation in belief (strong belief, partial belief, skepticism)	5	41.7

This understanding is reinforced by frequent references to dependence on water systems (10 out of 12 interviews), indicating that community survival is perceived as inseparable from the availability and behavior of water sources. Such dependence extends beyond material necessity, encompassing a broader relational configuration in which water is embedded within territorial and experiential frameworks. In this sense, water is not external to social life but constitutive of it.

Spiritual-territorial practices, including pagapu rituals and offerings (7 out of 12 interviews), further reinforce this understanding by situating water within systems of reciprocal engagement between community members and territorial entities. These practices are not merely symbolic, but constitute performative actions through which relationships with water are maintained and reproduced. Similarly, ontological narratives involving sirens, sacred animals, or enchanted places (8 out of 12 interviews) contribute to shaping how water sources are understood and engaged, embedding hydrosocial relations within a broader cosmological order.

**Figure 1:**
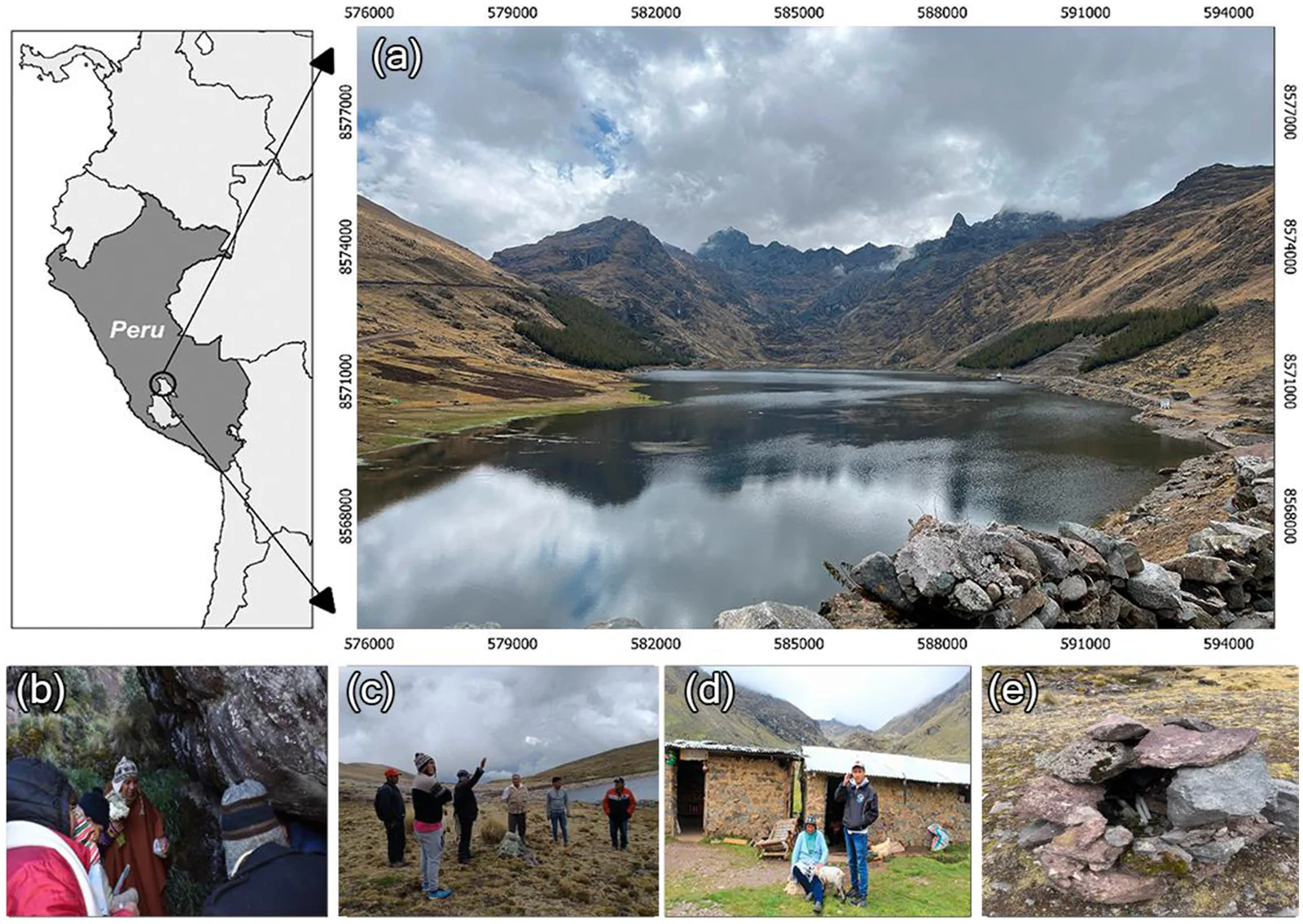
Hydrosocial territory and relational practices in the Razuhuillca watershed. **(a)** Location and landscape of the Razuhuillca lagoon within the Peruvian Andes. **(b)** *Pagapu* ritual (offering) performed for Apu Razuhuillca, expressing reciprocal relations between community and territorial entity. **(c)** Local guardians of the lagoons, reflecting collective responsibility for the protection and care of water bodies. **(d)** *In situ* interview with community members, illustrating lived experiences and relational understandings of water. **(e)** Stone-based offering associated with good fortune and the care of Apu Razuhuillca. Together, these images illustrate the material, ritual, and experiential dimensions through which hydrosocial relations are enacted and sustained.

Figure 1 provides visual evidence of these relational dynamics. The hydrosocial landscape of the Razuhuillca watershed (Figure 1a) illustrates the material configuration through which water sustains life, while ritual practices (Figure 1b) and collective guardianship (Figure 1c) demonstrate how these relations are enacted and maintained. The *in situ* interview (Figure 1d) highlights how such understandings are articulated through lived experience, and the stone-based offering (Figure 1e) reflects the material expression of engagement with the Apu. Together, these elements show that hydrosocial relations are enacted through the intersection of discourse, practice, and material interaction.

Importantly, while this relational understanding is widely shared, the presence of heterogeneous belief (5 out of 12 interviews) indicates that these positions are not uniform. Differences in the degree of belief or engagement suggest that hydrosocial relations operate within a dynamic field of interpretation rather than a homogeneous framework.

Overall, these findings indicate that water, territory, and nonhuman entities are not external objects of social action, but constitutive components of a relational ontology of existence. Hydrosocial relations thus emerge as the foundational condition through which life is understood and sustained, providing the basis for examining how subjectivity is constituted within this relational field.

### 3.2 Distributed agency and moral regulation

Building on the recognition of water as a condition of existence, the empirical material indicates that hydrosocial relations are structured through a distributed configuration of agency in which nonhuman entities actively participate in the regulation of environmental and social life. In particular, Apu Razuhuillca emerges not merely as a symbolic figure, but as an agentive territorial entity understood as capable of influencing water availability, ecological conditions, and human well-being.

This attribution of agency, observed in 9 out of 12 interviews (Table 1), reflects an ontological position in which causality is not restricted to human action. Participants repeatedly described the Apu as capable of “deciding” whether water flows are maintained or diminished, suggesting that environmental processes are interpreted as contingent upon relational dynamics involving both human and nonhuman actors. In this configuration, agency is distributed across a relational field that includes the mountain, water sources, and the community, challenging conventional distinctions between subject and object.

This distributed configuration is closely intertwined with a system of moral regulation governing interactions within the hydrosocial field. References to moral regulation appear in 8 out of 12 interviews, where water availability is interpreted in relation to human conduct, particularly respect, reciprocity, and adherence to ritual obligations. Acts perceived as disrespectful toward the Apu—such as neglecting offerings or failing to observe appropriate conduct in sacred sites—are associated with negative outcomes, including water scarcity, illness, or misfortune.

These interpretations point to a form of moral ecology in which environmental dynamics are embedded within ethical relationships. Rather than operating as external constraints, moral norms are integral to hydrosocial relations, shaping how individuals and communities engage with water and territory. Reciprocity, in particular, structures expectations of balanced interaction between humans and nonhuman entities, enacted through practices such as pagapu rituals and offerings.

Importantly, these relations are not articulated only at the level of discourse, but enacted through concrete practices. Ritual actions observed during fieldwork (Figure 1b) constitute moments in which relationships between community members and territorial entities are actively renewed, while the role of local guardians (Figure 1c) reflects collective responsibility for maintaining water sources. These practices demonstrate how moral regulation extends beyond individual behavior to encompass communal forms of care and stewardship.

At the same time, variation across participants introduces differences in how distributed agency and moral regulation are interpreted. While some express strong conviction regarding the agency of the Apu and the necessity of ritual practices, others adopt more ambivalent or skeptical positions. This variation highlights the dynamic and negotiated character of the hydrosocial field, in which different degrees of adherence coexist.

Overall, these findings suggest that hydrosocial relations are structured through an interplay between distributed agency and moral regulation. The Apu, water, and community form a relational assemblage in which environmental processes, ethical conduct, and social life are mutually constitutive. Within this configuration, individuals are positioned not as autonomous actors, but as participants in a network of obligations and relations that extend beyond the human domain, providing a foundation for the subsequent analysis of subject formation.

### 3.3 Mechanisms of relational self-formation

Building on the distributed configuration of agency and moral regulation described above, the analysis identifies a set of interrelated mechanisms through which hydrosocial relations are translated into forms of subjectivity. Rather than emerging as an inherent attribute of the individual, the self appears as a relational achievement produced through the interaction of discursive, experiential, and practical processes embedded within the hydrosocial field.

As summarized in Table 2, five key mechanisms structure this process: discursive enactment, distributed agency, moral embedding, experiential validation, and territorial practice. These mechanisms do not operate independently but are mutually reinforcing, forming a dynamic configuration through which subjectivity is continuously constituted.

**Table 2:** Mechanisms of relational hydrosocial self-constitution.

Mechanism	Analytical description	Empirical evidence	Illustrative example
Discursive enactment	Language constructs water and territory as living relational entities	Recurrent statements linking water to life and existence	“Water is life… without water there is no life”
Distributed agency	Agency attributed to non-human entities (Apu, mountain, lagoon)	Narratives of decision-making and control by the Apu	“The Apu decides if there will be water or not”
Moral embedding	Water relations structured through ethics of respect, reciprocity, and punishment	Accounts of offense, ritual obligation, and consequences	“If you don't respect it, the Apu gets offended and water disappears”
Experiential validation	Lived experiences reinforce relational ontology	Stories of illness, death, environmental change	Accounts of people getting sick or disappearing
Territorial practice	Subjectivity enacted through everyday and collective practices	Irrigation, livestock, rituals, territorial defense	Pagapu rituals and collective mobilization against mining

Discursive enactment constitutes a primary mechanism through which relational understandings are articulated and reproduced. Recurrent expressions such as “water is life” do not merely describe reality but function as performative statements that construct water and territory as living entities embedded within social existence. Through language, participants situate themselves within a relational ontology in which human life is inseparable from hydrosocial processes.

This discursive dimension is closely intertwined with distributed agency. As discussed in previous sections, participants consistently describe the Apu and other territorial entities as capable of influencing environmental conditions and human outcomes. This attribution extends the field of agency beyond the human, positioning individuals as participants within a relational system rather than as autonomous actors.

Moral embedding further structures this configuration by integrating ethical principles into hydrosocial interactions. Norms of respect, reciprocity, and obligation define appropriate behavior toward water and the Apu, linking environmental outcomes to moral conduct. These moral frameworks operate not as external rules but as constitutive elements of relational life, shaping how individuals perceive their responsibilities and position themselves within the hydrosocial field.

A crucial mechanism reinforcing these dynamics is experiential validation. Lived experiences, such as illness, environmental change, or unexpected events, are interpreted through relational frameworks that confirm the agency of nonhuman entities and the relevance of moral norms. These experiences provide empirical grounding for relational understandings, transforming them into embodied knowledge that guides action and reinforces collective interpretations.

Finally, territorial practice anchors subjectivity within everyday and collective activities. Irrigation, livestock management, ritual offerings, and participation in territorial defense are not merely economic or political actions, but practices through which individuals enact and reproduce their relationships with water and territory. Through these practices, the self is continuously constituted as part of a broader relational system linking community, environment, and sacred entities.

**Figure 2:**
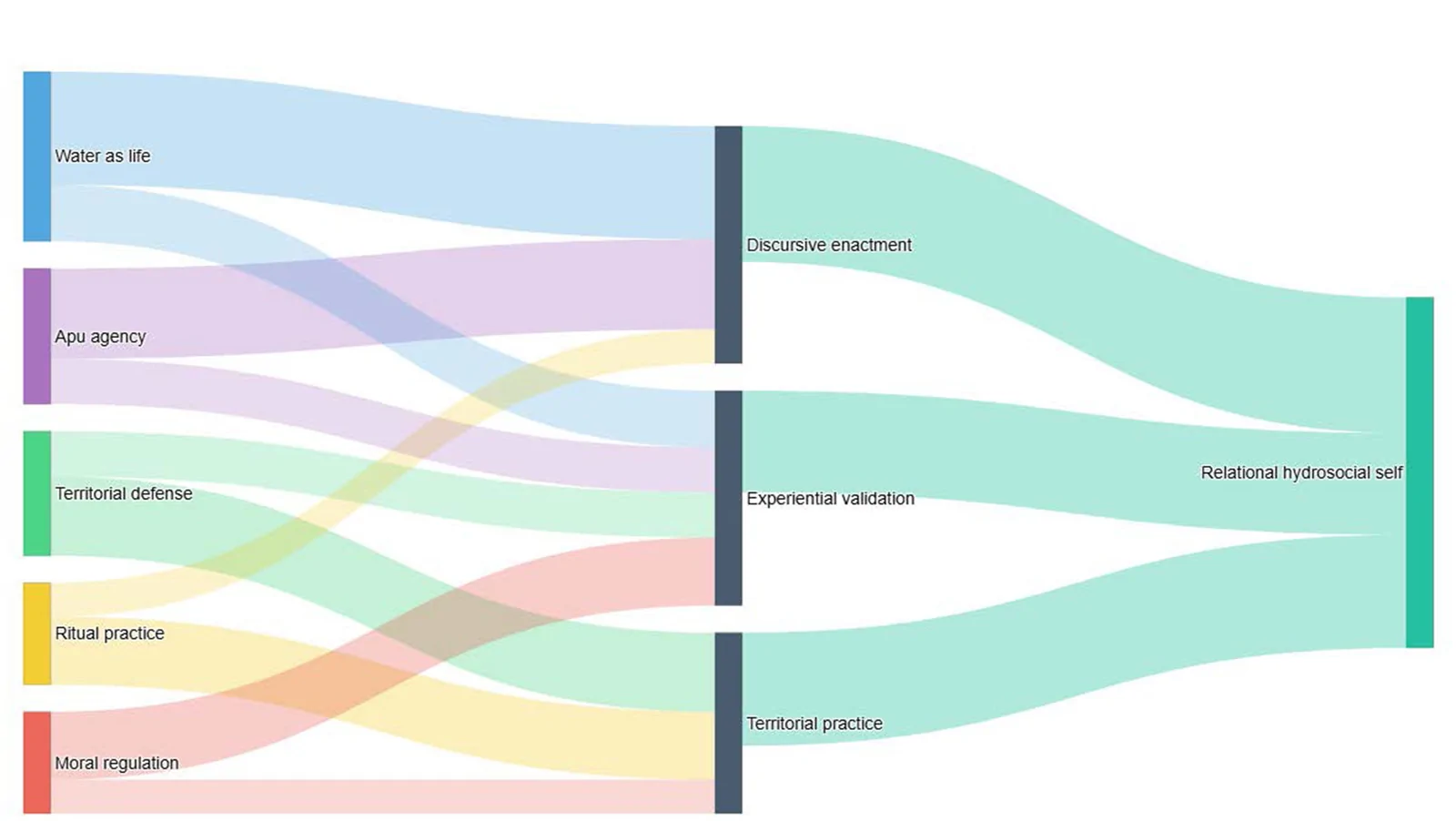
From hydrosocial relations to the constitution of the relational self.

Figure 2 provides a schematic synthesis of these processes. For analytical clarity, the figure emphasizes three integrative dimensions, discursive enactment, experiential validation, and territorial practice, which condense the broader set of mechanisms identified in Table 2. Together, these dimensions illustrate how hydrosocial relations are translated into processes of subject formation, giving rise to a relational hydrosocial self.

Taken together, these findings indicate that the constitution of the self in this context cannot be adequately understood through models centered on individual autonomy. Instead, subjectivity emerges through a relational configuration in which language, experience, practice, and moral engagement operate as interconnected mechanisms. The relational hydrosocial self can thus be understood as a distributed, processual, and territorially embedded form of subjectivity grounded in ongoing interactions between humans, water systems, and nonhuman entities.

### 3.4 Pathways and variation in ontological positioning

While the previous sections have outlined the relational configuration of hydrosocial subjectivity, the empirical material also indicates important variation in how these relations are interpreted and enacted. Rather than assuming a homogeneous ontological framework, participants display different degrees of commitment to relational understandings of water, territory, and nonhuman agency.

As shown in Table 3, three main ontological positions can be identified: strong relational ontology, partial or negotiated belief, and skeptical or modern stance. Half of the participants (6 out of 12 interviews) articulate a strong relational ontology, characterized by explicit and consistent attribution of agency to Apu Razuhuillca and a firm belief in its role in regulating water flows and environmental conditions. Statements such as “the Apu decides everything” reflect a coherent relational framework in which human life is understood as embedded within a network of reciprocal relations involving nonhuman entities.

**Table 3:** Variation in ontological positioning among participants.

Position type	Description	Frequency	Illustrative statement
Strong relational ontology	Explicit belief in Apu as active agent controlling water	6	“The Apu decides everything”
Partial/negotiated belief	Ambivalent or conditional belief in Apu agency	4	“It could be the Apu… maybe”
Skeptical/modern stance	Rejection or distancing from traditional beliefs	2	“We don't believe much in those things now”

A second group (4 out of 12 interviews) exhibits a more ambivalent or negotiated position. Participants in this category acknowledge the possibility of nonhuman agency but express uncertainty or conditional belief. Statements such as “it could be the Apu… maybe” suggest a hybrid configuration in which relational ontologies coexist with alternative explanatory frameworks, including more instrumental or naturalistic interpretations of environmental processes. This negotiated position reflects the coexistence of multiple epistemic orientations within the same hydrosocial context.

Finally, a smaller group (2 out of 12 interviews) adopts a skeptical or more explicitly modern stance, distancing themselves from relational interpretations and attributing water dynamics primarily to natural or technical factors. However, even in these cases, complete detachment from relational frameworks is not fully observed, as such positions remain situated within a broader social and territorial environment in which relational understandings continue to circulate.

**Figure 3:**
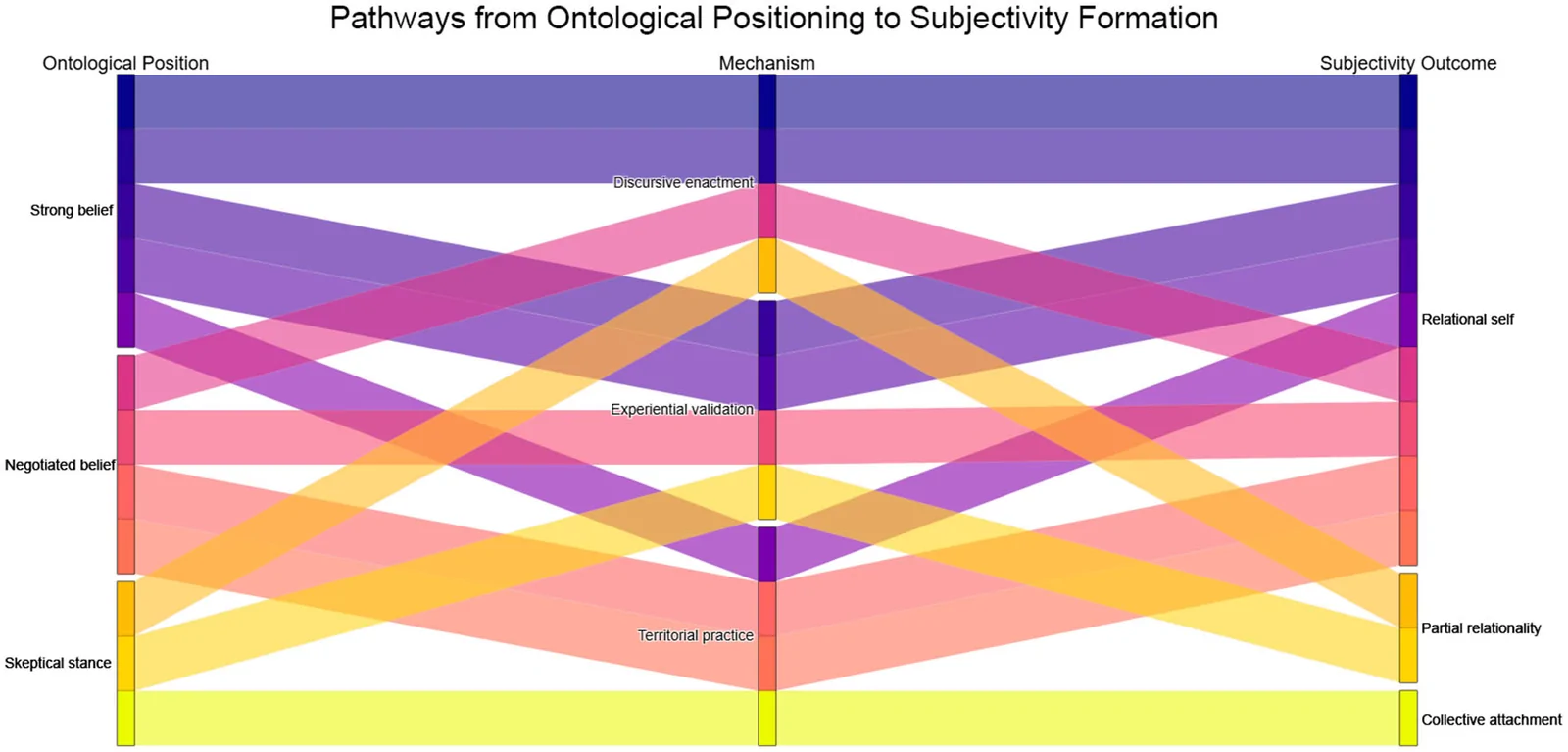
Pathways from ontological positioning to subjectivity formation.

Figure 3 provides a schematic representation of how these different ontological positions relate to pathways of subjectivity formation. The diagram illustrates how varying degrees of ontological commitment are associated with different configurations of engagement across key mechanisms such as discursive enactment, experiential validation, and territorial practice. The flows represented are analytical rather than statistical, and are intended to capture patterned relations identified in the qualitative material.

Participants with strong relational ontologies tend to exhibit more direct and consistent connections across these mechanisms, contributing to the consolidation of a relational hydrosocial self. In contrast, negotiated positions display more heterogeneous pathways, with partial or conditional engagement across mechanisms. Skeptical stances, while less directly aligned with relational mechanisms, still intersect with territorial practices and collective dynamics, indicating that subjectivity formation remains shaped by the broader hydrosocial field.

These findings indicate that relational subjectivity is not a fixed or uniform condition, but a dynamic and differentiated process shaped by varying degrees of ontological commitment. Importantly, variation does not imply fragmentation, but rather highlights the flexibility and resilience of hydrosocial relations as a field in which multiple forms of engagement coexist.

This variability reinforces the analytical value of the relational hydrosocial self as a concept that captures not a singular identity, but a spectrum of subject positions constituted through different configurations of belief, practice, and experience. By explicitly accounting for variation, the analysis avoids essentializing Indigenous or rural perspectives and instead emphasizes the negotiated and context-dependent character of relational ontologies.

Although the study was not designed to systematically compare social groups, preliminary differences emerged across participants. Older residents and individuals more directly involved in irrigation, livestock management, and territorial care tended to express stronger relational commitments to Apu Razuhuillca and hydrosocial reciprocity. In contrast, younger participants and those with greater exposure to external institutions more often articulated negotiated or partially skeptical positions. These patterns suggest that the relational hydrosocial self is internally differentiated and shaped by diverse life trajectories and forms of territorial engagement. These tendencies should be interpreted cautiously as qualitative patterns rather than systematic group differences given the exploratory design and limited sample size.

### 3.5 The emergence of the relational hydrosocial self

The preceding analysis indicates that hydrosocial relations do not merely shape environmental practices or cultural meanings, but actively participate in the constitution of subjectivity. Building on the identified mechanisms and the observed variation in ontological positioning, this section proposes the relational hydrosocial self as an empirically grounded form of subject formation.

Rather than conceiving the self as an autonomous and bounded entity, the findings suggest that subjectivity in the Razuhuillca watershed emerges through ongoing interactions among humans, water systems, and nonhuman territorial entities. The self does not precede these relations, but is constituted through them, taking form within a network of dependencies, obligations, and reciprocal engagements that extend beyond the human domain.

Three interrelated features characterize the relational hydrosocial self. First, it is distributed, in the sense that agency is not located exclusively within individuals but is shared across human and nonhuman actors. The Apu, water sources, and the broader landscape are not passive contexts, but participants in shaping outcomes, decisions, and experiences. Individuals therefore act not in isolation, but as part of a relational assemblage in which agency is co-produced.

Second, the relational hydrosocial self is morally embedded. Hydrosocial relations are structured through ethical frameworks that define appropriate conduct toward water and territorial entities, linking environmental conditions to practices of respect, reciprocity, and obligation. Subjectivity is thus inseparable from moral engagement, as individuals understand themselves in relation to the responsibilities they hold within the hydrosocial field.

Third, the self is territorially enacted through practice. Everyday activities such as irrigation, livestock management, ritual offerings, and participation in collective actions are not merely instrumental behaviors, but sites of subject formation. Through these practices, individuals reproduce their relationships with water and territory, embedding their identities within specific spatial and ecological contexts.

Importantly, the relational hydrosocial self is not a homogeneous or static configuration. As shown in the variation of ontological positioning, different degrees of belief and engagement produce diverse pathways through which subjectivity is constituted. This variability underscores the processual nature of the self, which emerges through shifting configurations of discourse, experience, and practice rather than through fixed cultural templates.

Taken together, these findings indicate that the relational hydrosocial self can be understood as a distributed, morally embedded, and territorially enacted form of subjectivity grounded in hydrosocial relations. Rather than representing a cultural particularity, it constitutes an analytically significant configuration that provides empirical grounding for rethinking dominant assumptions about the nature of the self.

## 4 Discussion

### 4.1 Rethinking the self beyond autonomy

The dominant conception of the self in social theory has been historically grounded in assumptions of autonomy, boundedness, and individual agency, positioning the subject as the primary locus of action while treating social and environmental contexts as external conditions. The findings of this study challenge this assumption by suggesting that subjectivity can emerge through relational configurations that extend beyond the individual to include ecological and nonhuman actors as constitutive participants.

Rather than operating as an independent unit, the self observed in the Razuhuillca watershed is constituted through ongoing interactions with water systems, territorial entities, and collective practices, challenging the ontological separation between subject and environment that underpins much of Western social theory. While relational sociology has shown that actors emerge through networks of interaction rather than pre-existing entities (Emirbayer, 1997), it has largely remained focused on human-to-human relations. The empirical material presented here supports the argument that subject formation is not only intersubjective but also hydrosocial and more-than-human, involving water, landscape, and territorial entities as active components of relational life.

This extension has important implications for critiques of methodological individualism. As Dépelteau (2008) argues, social reality is constituted through relations rather than discrete entities. The Razuhuillca case reinforces this claim, while suggesting that such relational processes are not limited to human interaction but are embedded in socio-ecological configurations in which nonhuman entities participate in shaping action, responsibility, and experience. In this sense, relationality extends beyond the social to include hydrosocial and territorial dimensions.

This perspective also contributes to process-oriented theories of the self that reject static notions of identity. Subjectivity is continuously produced through engagement with dynamic environments and shifting relational fields (Abbott, 2016), which in this case are materially and ecologically grounded. Rather than being merely situated within a social context, the self is constituted through interactions with water flows, landscape features, and nonhuman entities such as the Apu.

Taken together, these findings suggest that the self may be more productively understood beyond autonomy and bounded individuality as a distributed, processual, and ecologically embedded form of subjectivity. In this configuration, water, territory, and nonhuman entities are not external to social life but participate in the constitution of the self, challenging dominant theoretical models and extending relational approaches to include hydrosocial relations that integrate social, ecological, and cosmological dimensions.

### 4.2 Hydrosocial relations as ontological foundations

The findings of this study suggest that hydrosocial relations should be understood not merely as socio-environmental interactions, but as constitutive elements of distinct ontological frameworks. Rather than representing cultural interpretations of a shared reality, the relational configurations observed in the Razuhuillca watershed point to the enactment of a mode of existence in which water, territory, and nonhuman entities are integral to social life.

This perspective resonates with developments in the ontological turn, which argue that collectives do not simply hold different beliefs about the world, but actively constitute different realities through their practices and relations (Viveiros de Castro, 2015; Holbraad and Pedersen, 2017). In this sense, the attribution of agency to Apu Razuhuillca, the moral regulation of water flows, and the performance of ritual practices operate not as symbolic representations, but as ontological statements that structure reality as lived and experienced.

However, while political ontology has demonstrated the existence of ontological plurality, it has been less explicit about how such configurations shape the constitution of subjectivity. Existing work has primarily focused on how worlds are enacted and come into conflict, particularly in relation to territory, resources, and governance (Escobar, 2018; Blaser, 2013; de la Cadena, 2015). The findings presented here suggest that these configurations not only organize worlds, but may also produce particular forms of selfhood.

Within this framework, water cannot be reduced to a natural resource governed solely by physical processes. Instead, it emerges as a relational entity embedded in networks of reciprocity, obligation, and moral engagement. This aligns with approaches that conceptualize nature as a field of relations in which humans and nonhumans co-constitute each other (Descola, 2013), while extending their implications to the constitution of the self.

The political implications of this shift are particularly visible in contexts of hydrosocial conflict. As de la Cadena (2015) argues, the presence of entities such as mountains or spirits generates “ontological openings” where different realities intersect. The empirical evidence presented here supports this view, while suggesting that such conflicts are also sites where forms of subjectivity are enacted, negotiated, and defended through hydrosocial relations.

Understanding hydrosocial relations as ontological foundations has direct implications for how subjectivity is conceptualized. If water, territory, and nonhuman entities are constitutive of reality, the self cannot be understood independently of these relations, but emerges within an ontological field in which human and nonhuman elements are mutually constitutive. In this sense, the relational hydrosocial self may be approached not simply as a cultural variation, but as an analytically significant form of subjectivity grounded in a specific configuration of existence.

Taken together, these findings suggest that hydrosocial relations are foundational to the co-constitution of reality and subjectivity, extending political ontology beyond the analysis of plural worlds toward a more explicit theorization of how selves are formed within them.

### 4.3 Distributed agency and more-than-human subjectivity

The empirical findings of this study challenge not only the autonomy of the individual subject, but also the assumption that agency is exclusively human. The attribution of decision-making capacity to Apu Razuhuillca, together with the recognition of water and landscape as active participants in social life, points toward a distributed configuration of agency that extends beyond the human domain. This challenges anthropocentric models of subjectivity and supports the need to conceptualize agency as relational, hydrosocial, and more-than-human.

From the perspective of actor-network theory, agency is not an intrinsic property of individuals but an effect of associations among heterogeneous actors, both human and nonhuman (Latour, 2005). In this view, entities such as mountains, water systems, and infrastructures are not passive backdrops but active mediators that shape outcomes and possibilities for action. The Razuhuillca case aligns with this perspective, as participants interpret environmental processes, including the presence or absence of water, as contingent upon the actions of nonhuman entities.

At the same time, the findings suggest an important extension. While actor-network theory redistributes agency across human and nonhuman actants, it has been less explicit about how such distributed agency is experienced and incorporated into subject formation. In the Razuhuillca watershed, nonhuman agency is not only an analytical effect of relational networks but also a lived and morally consequential dimension of social life. The Apu is not simply an actant, but a territorial entity that organizes obligation, responsibility, and forms of conduct.

This perspective is further supported by posthumanist approaches that emphasize the entanglement of humans and nonhuman worlds. Haraway (2008) conceptualizes existence as constituted through “naturecultures,” while Whatmore (2002) highlights “hybrid geographies” in which humans, nonhumans, and environments are co-implicated in social life. The empirical material presented here aligns with these perspectives, while specifying how such entanglements are translated into forms of subjectivity grounded in hydrosocial relations.

In this context, distributed agency has direct implications for subject formation. Individuals are not positioned as autonomous actors, but as participants within a relational field that includes water flows, territorial entities, and cosmological forces. Subjectivity thus emerges as a situated and embodied engagement with a world in which nonhuman entities actively shape possibilities for action, obligation, and experience.

Importantly, the extension of agency beyond the human does not imply the dissolution of responsibility, but its reconfiguration. Responsibility becomes relational and morally embedded, grounded in interdependence and enacted through practices such as ritual offerings and expressions of respect toward the Apu. These practices illustrate how responsibility is negotiated within a field that includes both human and nonhuman actors.

By situating subjectivity within a more-than-human framework, the findings contribute to debates on the decentering of the human in social theory. At the same time, they suggest that distributed agency is not only a feature of socio-material networks, but may also be understood as a constitutive dimension of the self. The relational hydrosocial self thus emerges as a form of subjectivity in which agency, responsibility, and identity are co-produced through interactions that traverse social, ecological, and cosmological domains.

### 4.4 Moral ecology and the ethical constitution of the self

Beyond the distribution of agency across human and nonhuman actors, the findings of this study suggest that hydrosocial relations are structured through moral and ethical frameworks. Interactions with water, territory, and Apu Razuhuillca are not governed solely by practical or ecological considerations, but by norms of respect, reciprocity, and obligation that define appropriate modes of engagement within the hydrosocial field. This indicates that subjectivity is not only relational but also ethically constituted.

This perspective can be understood through the notion of moral ecology, which emphasizes that environmental relations are embedded within systems of value and ethical responsibility (Berkes, 2012). In such frameworks, ecological processes are interpreted through culturally grounded principles that link human conduct with environmental outcomes. The Razuhuillca case extends this understanding by suggesting that moral regulation is not only a guiding principle for environmental management, but also a constitutive dimension of how reality is experienced and enacted.

Reciprocity plays a central role in this configuration. As Gudeman (2001) suggests, economic and social life in many contexts is structured through relations of mutual obligation rather than individual accumulation. In the hydrosocial relations observed here, practices such as pagapu rituals operate as concrete enactments of reciprocity, through which relationships between humans and nonhuman entities are maintained and renewed.

This ethical configuration resonates with broader discussions on cosmopolitics, where multiple forms of existence are recognized as participants in shared worlds requiring negotiation and care (Stengers, 2010). In this sense, the Apu is not merely an object of belief, but a participant in a moral and political order that organizes responsibility and conduct.

Importantly, this ethical dimension reshapes how responsibility is understood. Rather than being individualized, responsibility is distributed across a network of relations in which actions have consequences that extend beyond the human sphere. Narratives linking disrespect toward the Apu with scarcity or misfortune illustrate how moral conduct is intertwined with environmental conditions, reinforcing the inseparability of ethics and ecology.

Taken together, these findings suggest that the relational hydrosocial self is constituted not only through interactions and practices, but through ethical commitments that position individuals within a network of reciprocal relations. Subjectivity thus emerges as a moral process grounded in interdependence, challenging models of the self as morally autonomous and highlighting an ethics of relational responsibility.

### 4.5 Variation, plurality, and non-essentialism

While the findings of this study highlight the presence of relational hydrosocial subjectivities, they also indicate that these configurations are neither uniform nor equally shared among participants. The empirical material reveals variation in ontological positioning, ranging from strong relational commitments to more ambivalent or skeptical orientations. Recognizing this variation is essential to avoid essentializing local knowledge systems or representing Andean subjectivities as internally homogeneous.

This point is analytically important because relational ontologies do not operate as fixed cultural templates, but are enacted, negotiated, and sometimes contested within the hydrosocial field. As the results show, some participants consistently attribute agency to Apu Razuhuillca and interpret water relations through reciprocal moral obligations, while others express partial belief, uncertainty, or more naturalistic interpretations of environmental processes. These differences do not place actors inside or outside the relational field, but reflect varying degrees and pathways of engagement with hydrosocial relations.

Although the present study was not designed to systematically compare social categories, some variation appeared to be associated with differences in life experience, degree of involvement in water-related practices, and exposure to external institutions. Future research could examine more explicitly how generational trajectories, gendered responsibilities, socioeconomic position, and patterns of territorial engagement influence pathways of hydrosocial subject formation. Such an approach would further illuminate the internal diversity of relational ontologies while avoiding assumptions of cultural homogeneity.

Such variation strengthens rather than weakens the argument of this article. It suggests that the relational hydrosocial self should not be understood as a singular or essential identity, but as a spectrum of subject positions constituted through different configurations of discourse, experience, and practice. This allows the analysis to account for plurality without reducing it to fragmentation, and to recognize that relational subjectivity can remain socially significant even where belief is partial or unstable.

This emphasis on variation also responds to broader critiques of essentialism in the study of Indigenous and rural worlds. Rather than assuming a coherent and unchanging ontology, the Razuhuillca case points to a dynamic field in which multiple epistemic orientations coexist. Relational and more naturalistic interpretations do not appear as mutually exclusive blocks, but as overlapping and unevenly articulated ways of engaging water, territory, and nonhuman agency.

The analytical consequence is that the relational hydrosocial self must be understood as processual, differentiated, and context-dependent. Its significance lies not in representing a universal form of subjectivity, but in revealing how forms of selfhood emerge through variable engagements with hydrosocial relations. By foregrounding plurality and non-essentialism, the study reinforces its theoretical claim while avoiding reductive representations of local worlds.

### 4.6 Theoretical contribution: the relational hydrosocial self

The main theoretical contribution of this article is not simply to apply relational or ontological approaches to an Andean case, but to address a specific and under-theorized problem across these literatures: the lack of a precise account of how subjectivity is constituted through relations in which ecological and nonhuman entities are not contextual but constitutive.

Existing scholarship has made significant advances in decentering the autonomous individual. Relational sociology has shown that actors emerge through networks of interaction, political ontology has demonstrated the plurality of worlds, and more-than-human approaches have redistributed agency across human and nonhuman entities. However, these perspectives have remained less explicit about how such relational, ontological, and more-than-human configurations are incorporated into the constitution of the self itself. In particular, water, territory, and nonhuman beings have often been treated as relations, ontologies, or actants, but less often as constitutive elements of subject formation.

The concept of the relational hydrosocial self addresses this gap by offering a more precise account of how identity, agency, and moral responsibility may be constituted through hydrosocial relations. It provides an analytical account of subject formation in which water systems, territorial practices, and nonhuman entities participate in the production of the self, rather than functioning only as external conditions, symbolic frameworks, or ontological backgrounds.

The relational hydrosocial self is best understood not as a claim to absolute conceptual novelty, but as a theoretical specification and synthesis that helps address this problem. Its contribution lies in making explicit the mechanisms through which subjectivity may be constituted across relational, ecological, and cosmological domains, thereby integrating insights that have remained only partially connected in existing approaches.

This contribution extends relational sociology in a specific way. While relational sociology has emphasized that actors are constituted through networks of interaction (Emirbayer, 1997), it has largely remained focused on human-to-human relations. The relational hydrosocial self expands this framework by showing how subjectivity may be constituted not only through social interaction, but through relations that include ecological processes, territorial configurations, and cosmological entities as active participants.

At the same time, the concept advances discussions in political ontology. Existing work has shown that different collectives enact distinct realities (Escobar, 2018; Blaser, 2013; de la Cadena, 2015). This article extends that insight by suggesting that such ontological configurations do not only organize worlds, but may also shape forms of subjectivity. The relational hydrosocial self thus shifts the analytical focus from ontological plurality alone toward the constitution of the self within those ontological fields.

The concept also contributes to more-than-human and posthuman approaches. While these perspectives have been effective in decentering the human and redistributing agency (Latour, 2005; Haraway, 2008), they have been less explicit about how distributed agency becomes internal to subject formation. The relational hydrosocial self addresses this gap by showing that distributed agency can be understood not only as a feature of socio-material networks, but also as a constitutive dimension of the self.

A key contribution of the concept lies in its specification of mechanisms. By identifying discursive enactment, distributed agency, moral embedding, experiential validation, and territorial practice as interrelated processes, the article offers a concrete account of how subjectivity may be produced through hydrosocial relations. This moves beyond abstract claims about relationality toward an empirically grounded explanation of subject formation.

The relational hydrosocial self can thus be analytically characterized through four interrelated dimensions: relationality, distributed agency, moral embedding, and territorial practice. These dimensions are not independent attributes, but mutually constitutive processes through which subjectivity is continuously produced.

Importantly, this concept is not proposed as a universal model of subjectivity, but as an analytically generalizable framework derived from a specific empirical context. Its significance lies in suggesting that forms of the self constituted through hydrosocial relations are not only articulated as cultural interpretations, but can also be approached as empirically observable configurations that challenge dominant assumptions of autonomy and bounded individuality.

By specifying how subjectivity may be constituted across social, ecological, and cosmological domains, the relational hydrosocial self contributes to the pluralization of social theory and provides a basis for rethinking the self in ways that are more attuned to the complexity of socio-ecological life.

### 4.7 Implications for environmental governance and conflict

The findings of this study have direct implications for environmental governance and the management of socio-environmental conflicts, particularly in contexts where multiple ontological frameworks coexist. Conventional approaches to water governance are typically grounded in technocratic and utilitarian models that conceptualize water as a resource to be allocated and managed. The evidence presented here suggests that such frameworks are analytically limited, as they do not fully account for the ways in which water, territory, and nonhuman entities are constitutive of social life and subjectivity.

In contexts where water is understood as a relational entity and where nonhuman actors such as Apu Razuhuillca are recognized as participants in social life, governance cannot be reduced to technical management alone. It must engage with competing ontological definitions of water, agency, and responsibility. As hydrosocial scholarship has shown, water governance is shaped by power relations, cultural meanings, and political struggles that extend beyond formal institutions (Boelens et al., 2016; Cardenas Morales et al., 2025c). The present findings extend this perspective by suggesting that governance also involves the recognition of different forms of subjectivity constituted through hydrosocial relations.

This ontological plurality becomes particularly visible in situations of conflict, such as disputes over mining concessions or water access. These conflicts are not only disagreements over resource distribution, but confrontations between different ways of organizing existence and responsibility. As Li (2014) argues, development interventions often fail to recognize such differences, leading to governance frameworks that marginalize local modes of existence. The Razuhuillca case suggests that ignoring relational ontologies may intensify conflict by disrupting systems of reciprocity and moral regulation that sustain hydrosocial relations.

Recognizing the relational hydrosocial self has implications for how participation and decision-making are conceptualized. If subjectivity is constituted through relations with water, territory, and nonhuman entities, governance processes cannot rely exclusively on individualistic models of participation. Instead, they must engage with collective and relational forms of agency, including ritual practices and territorial responsibilities. This implies expanding what counts as relevant knowledge and legitimate participation.

At the same time, incorporating relational ontologies into governance does not imply abandoning technical or scientific approaches, but reconfiguring them within more plural and context-sensitive frameworks. Recent debates on environmental governance emphasize the need for inclusive approaches that accommodate diverse knowledge systems and ontological perspectives (Kothari et al., 2019). The relational hydrosocial self contributes to this agenda by offering a framework for understanding how subjectivity, agency, and responsibility are configured relationally.

Based on the findings, three practical implications can be identified for environmental governance in hydrosocial conflict settings. First, governance frameworks should recognize local hydrosocial ontologies and relational understandings of water as legitimate components of environmental decision-making. Second, participatory processes should move beyond individual-centered consultation models and incorporate collective territorial responsibilities, customary practices, and community-based forms of environmental stewardship. Third, conflict-resolution mechanisms should combine technical assessments with intercultural and ontological dialogue capable of engaging different understandings of water, territory, and agency. These measures may contribute to more inclusive, context-sensitive, and socially legitimate governance arrangements.

More broadly, these findings suggest that effective environmental governance in contexts of socio-environmental conflict depends on the capacity to recognize and negotiate multiple ontologies of water, territory, and subjectivity. Governance thus becomes not only a matter of resource management, but of engaging with different configurations of reality and the forms of self that emerge within them. In this sense, the relational hydrosocial self highlights the need for governance approaches that are not only technically effective, but also ontologically inclusive and ethically grounded.

### 4.8 Limitations and scope conditions

This study has several limitations that should be acknowledged. First, it is based on a single case study in the Razuhuillca micro-watershed and is therefore intended for analytical generalization rather than statistical inference. While the findings provide a theoretically informed account of relational subject formation, their applicability to other contexts depends on the presence of comparable hydrosocial and ontological configurations.

Second, the qualitative design and relatively small sample size (*n* = 12) prioritize depth of interpretation over representativeness. Although thematic saturation was achieved, the results should be understood as capturing recurrent relational patterns rather than exhaustive variation across the population.

Third, field access constraints limited direct observation in high-altitude areas of the watershed, where certain ritual practices and interactions with territorial entities may be more intensively enacted. As a result, the analysis relies primarily on narratives and practices observed in more accessible areas, which may underrepresent spatial variation in hydrosocial relations.

These limitations do not undermine the analytical contribution of the study, but rather define its scope and highlight the need for further comparative and multi-sited research on relational forms of subjectivity in socio-ecological contexts.

Future research could examine whether similar mechanisms of hydrosocial subject formation emerge in other Andean watersheds, Indigenous territories, and socio-environmental conflict settings. Comparative and multi-sited studies would help assess the analytical transferability of the relational hydrosocial self and identify both common patterns and context-specific variations across different hydrosocial configurations. Such research would contribute to refining the scope conditions of the concept and strengthening its analytical generalization beyond the Razuhuillca case.

## 5 Conclusion

This study advances an alternative understanding of subjectivity by showing that the constitution of the self can emerge through hydrosocial relations rather than from autonomous individuality. Drawing on empirical evidence from the Razuhuillca watershed, it shows that identity, agency, and responsibility are configured through relational processes that integrate humans, water systems, and nonhuman territorial entities.

Beyond its empirical contribution, the article develops the concept of the relational hydrosocial self as a way of rethinking subject formation in socio-ecological contexts. This concept shifts the analytical focus from the individual as an isolated unit toward the self as a relational, processual, and more-than-human configuration. In doing so, it extends relational sociology beyond human-centered interaction, advances political ontology by linking ontological plurality to subject formation, and contributes to hydrosocial theory by suggesting that, in this case, water relations shape not only territory and governance, but also the formation of the self.

The findings suggest that relational forms of subjectivity can be understood as coherent and lived configurations that organize action, obligation, and responsibility. Recognizing these forms of subjectivity is therefore not only a theoretical concern, but also an important step for understanding socio-environmental conflicts in contexts characterized by ontological plurality.

At the same time, the study highlights the need for further research on the variability and transformation of relational subjectivities. Future work could examine how these forms evolve under conditions of environmental change, economic pressure, and institutional intervention, as well as how they interact with dominant governance frameworks across different socio-ecological settings.

More broadly, the relational hydrosocial self invites a reconsideration of what it means to be a subject in a context of ecological interdependence. If subjectivity is constituted through relations that traverse social, ecological, and cosmological domains, then the self cannot be fully captured as autonomous and bounded. Instead, it is more productively approached as a relational and ethically embedded process.

Reframing the self in these terms is not only a theoretical task, but may also contribute to the development of environmental governance approaches capable of engaging with the plurality of worlds, relations, and responsibilities that define contemporary socio-ecological life.

## Data Availability

The original contributions presented in the study are included in the article/Supplementary material, further inquiries can be directed to the corresponding authors.
