# Correction: Beyond the autonomous individual: hydrosocial relations and the constitution of the relational hydrosocial self in an Andean conflict

**DOI:** 10.3389/fsoc.2026.1995892

**Published:** 2026-09-25

**Authors:** Saríah Fanny Oré Gálvez, Alex Foyams Molina Linares, Jherry Lobaton Phocco Minaya, Alex Quispe Quispe, Solón Dante Carhuallanqui Ibarra, Juan Carlos Terres León, Rubén Ñaupari Molina, Crispin H. W. Barnes, Luis De Los Santos Valladares

**Affiliations:** 1 Escuela Profesional de Ingeniería Ambiental, Universidad Nacional Autónoma de Huanta, Ayacucho, Peru; 2 Escuela Profesional de Ingeniería Topográfica y Agrimensura, Facultad de Ciencias Agrarias Universidad Nacional del Altiplano Puno, Puno, Peru; 3 Escuela Profesional de Educación, Especialidad de Ciencias Sociales, Universidad Nacional de San Antonio Abad del Cusco, Cusco, Peru; 4 Universidad Nacional José María Arguedas, Andahuaylas, Peru; 5 Cavendish Laboratory, Department of Physics, University of Cambridge, Cambridge, United Kingdom

**Keywords:** Andean studies, distributed agency, hydrosocial territory, moral ecology, ontology, relational hydrosocial self, socio-environmental conflict, territorial subjectivity

Affiliation “Escuela Profesional de Ingeniería Ambiental, Universidad Nacional Autónoma de Huanta, Ayacucho, Peru” was erroneously assigned to author Alex Quispe Quispe. The correct affiliation for this author is “Escuela Profesional de Educación, Especialidad de Ciencias Sociales, Universidad Nacional de San Antonio Abad del Cusco, Cusco, Peru.”

The original version of this article has been updated.

