# Supplementary material for: Beyond the autonomous individual: hydrosocial relations and the constitution of the relational hydrosocial self in an Andean conflict
Source: Front Sociol. 2026 Sep 7;11:1856916. doi: 10.3389/fsoc.2026.1856916 (PMC13591766; doi:10.3389/fsoc.2026.1856916)
Supplement: Supplementary file 1 [file Table_1.DOCX]

Beyond the Autonomous Individual: Hydrosocial Relations and the Constitution of the Relational Hydrosocial Self in an Andean Conflict

Saríah Fanny Oré Gálvez ^a*^, Alex Foyams Molina Linares^a^, Jherry Lobaton Phocco Minaya ^b^, Alex Quispe Quispe ^a^, Solón Dante Carhuallanqui Ibarra ^a^, Juan Carlos Terres León ^d^ , Olga Karina Durand De La O ^a^, Cecilia Choque Pomasunco ^a^, Rubén Ñaupari Molina ^a*^,Crispin H.W. Barnes^e^, Luis De Los Santos Valladares^e,*^

^a^ Escuela Profesional de Ingeniería Ambiental, Universidad Nacional Autónoma de Huanta, Jr. Manco Cápac 497, Huanta, Ayacucho, Peru.

^b^ Escuela Profesional de Ingeniería Topográfica y Agrimensura, Facultad de Ciencias Agrarias Universidad Nacional del Altiplano Puno, Puno, Perú.

^c^ Escuela Profesional de Educación, Especialidad de Ciencias Sociales, Universidad Nacional de San Antonio Abad del Cusco, Cusco, Peru

^d^ Universidad Nacional José María Arguedas, Jr. Juan Francisco Ramos N.º 380, Andahuaylas, Apurímac, Perú.

^e^ Cavendish Laboratory, Department of Physics, University of Cambridge, 19 J.J. Thomson Ave., Cambridge CB3 0US, United Kingdom

Supplementary Material

Table S1. Interview sample characteristics

| **ID** | **Gender** | **Approx. Age** | **Role** | **Location** | **Date** |
| --- | --- | --- | --- | --- | --- |
| I1 | Male | 50s | Farmer | Razuhuillca | April 2026 |
| I2 | Male | 40s | Water user | Razuhuillca | April 2026 |
| I3 | Female | 60s | Community member | Razuhuillca | April 2026 |
| I4 | Male | 30s | Livestock farmer | Razuhuillca | April 2026 |
| I5 | Male | 20s | Local resident | Razuhuillca | April 2026 |
| I6 | Male | 60s | Elder | Razuhuillca | April 2026 |
| I7 | Mixed group | Various | Community | Razuhuillca | April 2026 |

Table S2. Codebook for qualitative analysis

| **Code** | **Definition** | **Example** |
| --- | --- | --- |
| Water as life | Water framed as essential for existence | “Without water we cannot live” |
| Total dependence | Community survival tied to water | “Huanta would die without water” |
| Apu agency | Mountain as decision-maker | “The Apu decides water availability” |
| Moral regulation | Water linked to ethical behavior | “If you offend it, it punishes you” |
| Ritual practice | Sacred practices regulating water relations | Pagapu, offerings |
| Ontological narratives | Mythical entities shaping reality | Sirens, sacred animals |

Table S3. Lexical frequency (supporting quantitative analysis)

| **Term** | **Frequency** |
| --- | --- |
| Water | High |
| Life | High |
| Apu | Medium–High |
| Mountain | Medium |
| Respect | Medium |
| Ritual | Medium |

Table S4. Co-occurrence patterns

| **Term 1** | **Term 2** | **Interpretation** |
| --- | --- | --- |
| Water | Life | Ontological dependence |
| Apu | Decide | Non-human agency |
| Water | Respect | Moral relation |
| Mountain | Protect | Territorial guardianship |

Figure S1.

Figure S2. Circular relational flow of hydrosocial subject-Formation

Figure S3. Hierarchical Composition of the relational Hydrosocial self
